# Cost-Effectiveness Analysis for the Treatment of Hyperphosphatemia in Predialysis Patients: Calcium-Based versus Noncalcium-Based Phosphate Binders

**DOI:** 10.1155/2018/2138528

**Published:** 2018-09-19

**Authors:** B. L. Goh, A. Soraya, A. Goh, K. L. Ang

**Affiliations:** ^1^Clinical Research Centre, Serdang Hospital, Kajang 43000, Malaysia; ^2^Azmi Burhani Consulting, Petaling Jaya 47820, Malaysia

## Abstract

**Background:**

Hyperphosphatemia in chronic kidney disease (CKD) patients is often treated with calcium carbonate (CaCO3) despite the fact that CaCO3 is associated with increased calcium load and potentially increased cardiovascular risk. Alternative treatments with noncalcium-based phosphate binders do not increase the calcium load but are more costly. This study analyzes the cost-effectiveness of sevelamer versus CaCO3 for the treatment of hyperphosphatemia in stage III-V predialysis CKD patients in Malaysia.

**Methods:**

A Markov decision model was adapted to simulate a hypothetical cohort of CKD patients requiring treatment for hyperphosphatemia. Survival was estimated by using efficacy data from the INDEPENDENT-CKD clinical trial. Cost data was obtained from Malaysian studies while health state utilities were derived from literature. Analysis was performed over lifetime duration from the perspective of the Ministry of Health Malaysia with 2013 as reference year.

**Results:**

In the base case analysis, sevelamer treatment gained 6.37 life years (5.27 QALY) compared to 4.25 life years (3.54 QALY) with CaCO3. At 3% discount, lifetime costs were RM159,901 ($48,750) and RM77,139 ($23,518) on sevelamer and CaCO3, respectively. Incremental cost-effectiveness (ICER) of sevelamer versus CaCO3 was RM47,679 ($14,536) per QALY, which is less than the WHO threshold of three times GDP per capita (RM99,395) per QALY. Sensitivity analyses, both using scenario sensitivity analysis and probabilistic sensitivity analysis, showed the result to be robust.

**Conclusions:**

Our study finds that sevelamer is potentially cost-effective compared to CaCO3, for the treatment of hyperphosphatemia in predialysis CKD III-V. We propose that sevelamer should be an option in the treatment of Malaysian predialysis patients with hyperphosphatemia, particularly those with high calcium load.

## 1. Introduction

Treatment of CKD imposes a substantial cost on health care budgets and middle-income countries (MIC) face significant challenges. In Malaysia, a middle-income country, an estimated 9.1% of the adult population suffered from CKD in 2013 [1] and this number is rising largely due to the increasing number of patients diagnosed with type 2 diabetes in recent years caused by changing lifestyles and dietary habits [2, 3]. CKD accounted for 27% of the total US Medicare budget in 2007 [4, 5]. In 2005, Malaysia had spent an estimated RM379 million to provide dialysis services for 13,355 patients [6]. By 2016, the number of CKD Vd patients in Malaysia has tripled to 39,711 [7].

One of the complications that develop in CKD patients is hyperphosphatemia. Studies have shown that elevated serum phosphate increases risk of mortality and cardiovascular disease (CVD) even for patients with early CKD. In fact, high serum phosphate levels are also associated with more rapid decline in renal function [8–10]. Kidney Disease: Improving Global Outcomes (KDIGO) guidelines recommended maintaining serum phosphate in the normal range (0.81 to 1.45 mmol/l) for patients with renal failure, CKD stage III to V (CKD-ND) [11].

Calcium-based binders (CBBs) such as calcium carbonate (CaCO3) are commonly used since they are widely available and cheap. However, clinical studies have shown that the use of oral calcium in the form of supplements or phosphate binders may lead to arterial calcification and an increased risk of mortality and CVD events [12–15]. The KDIGO guidelines recommended restricting the dose of CBBs for CKD patients in the presence of arterial calcification [11]. The INDEPENDENT-CKD trial which was performed among 212 patients (107 assigned to sevelamer and 105 assigned to CaCO3) reported that treatment with sevelamer in CKD-ND patients was associated with reductions in mortality and dialysis initiation [16]. Despite concerns regarding the long-term safety of CBBs, CaCO3 is still the first-line treatment for CKD patients in many low and MIC like Malaysia as they cost less than non-CBBs. Data from the Malaysian National Renal Registry indicated that, in 2016, more than 95% of CKD Vd patients were prescribed with CaCO3, and only 17% have a serum phosphate level of less than 1.3 mmol/l, indicating achievement of target based on the KDIGO guidelines was far from satisfactory [7].

There was evidence that non-CBB like sevelamer was cost-effective in two European countries; the value of this information is limited in the local setting due to differences in health systems and cost structures [17–19]. Thus, this study was conducted to determine the cost-effectiveness of sevelamer compared to CaCO3 for treatment of hyperphosphatemia in CKD-ND patients in Malaysia.

## 2. Methods

We performed an economic evaluation by adapting the previously developed decision-analysis model: the Markov decision model was adapted to simulate a hypothetical cohort of CKD patients requiring treatment for hyperphosphatemia using available Malaysian data [17]. Decision analysis is an approach for decision making taking into account uncertainty and evaluation of the consequences of alternative courses of action in terms of their costs and outcomes. Markov models are a type of decision-analysis model which are used to analyse uncertain processes, such as chronic diseases in which costs and outcomes occur over a long period of time [20]. Markov type decision models have been widely used in cost-effectiveness analysis including the use of sevelamer in dialysis and CKD-ND patients, as well as other chronic kidney disease interventions [17, 21, 22].

### 2.1. Model Overview

**Figure 1:**
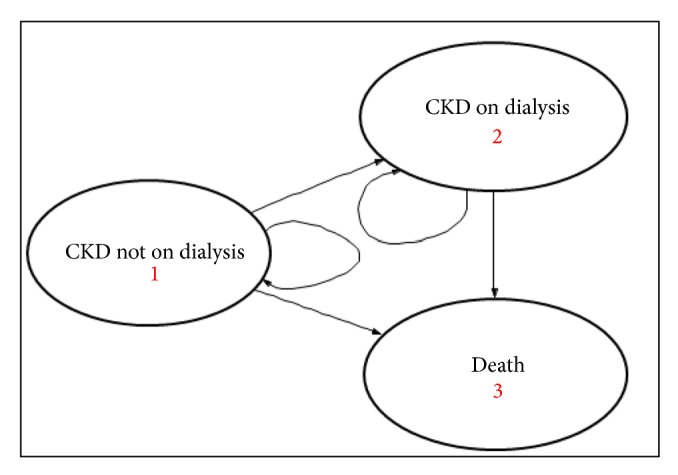
State transition diagram of the CLEAR-CKD model.

The Markov decision model simulates the progression from predialysis CKD to dialysis and death in a hypothetical cohort of CKD-ND patients treated with sevelamer carbonate or CaCO3 for hyperphosphatemia. The model structure is illustrated in Figure 1. The cohort entered the model in the health state “CKD not on Dialysis” and in subsequent one-year cycles, patients could remain alive without dialysis or transition to dialysis or death. Each cycle spent in the health states of “CKD not on Dialysis” or “CKD on dialysis” was associated with an amount of cost and quality of life. In the event of death, the simulated patient no longer incurs any cost nor gains any heath outcome. The total duration spent by the cohort in each of the health states was aggregated to obtain the total cost and quality-adjusted life years (QALY) accrued on treatment with sevelamer or CaCO3, respectively. Baseline characteristics of the trial subjects are shown in Table 1. The data inputs are summarized in Table 2.

### 2.2. Efficacy Data

**Figure 2:**
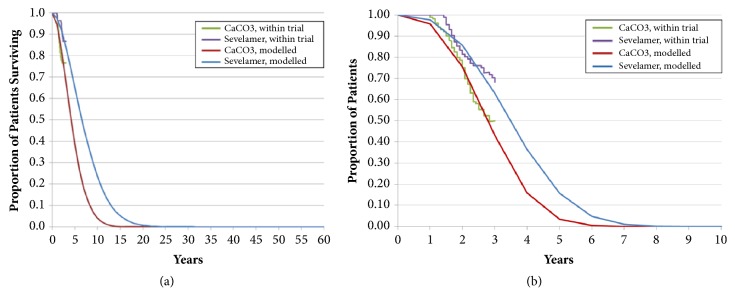
(a) Patient survival projected the from INDEPENDENT-CKD trial. Source: Di Iorio 2012, Cornerstone Research Group 2012. (b) Dialysis initiation projected from the INDEPENDENT-CKD trial. Source: Di Iorio 2012, Cornerstone Research Group 2012.

Transition probabilities between model health states were derived from the survival and dialysis initiation endpoints from the INDEPENDENT-CKD trial which followed patients for a duration of 3 years or until death (Figures 2(a) and 2(b)) [16].

### 2.3. Health Utility Data

Health utility data inputs in the model required data for the dialysis and predialysis health states. For the quality of life (QOL) of dialysis, we used QOL data of Malaysian dialysis patients [23]. The utility score associated with a predialysis health state was based on a study by Gorodetskaya I et al. [24]. The base case utility input values are listed in Table 2.

### 2.4. Resource Use and Costs

The perspective of the analysis was that of the provider, i.e., Ministry of Health Malaysia (MOH). Direct medical costs of drugs and dialysis incurred by the MOH were included in the analysis. The costs of other medical resources, i.e., hospitalization, concomitant drugs, treatment of adverse events (AE), and indirect costs (out-of-pocket expenses, productivity losses), were excluded from the analysis.

The medication costs of sevelamer and CaCO3 were calculated by multiplying the average daily drug dose reported in the INDEPENDENT-CKD trial with the latest available unit prices of sevelamer and generic CaCO3 in Malaysia. The cost per gram of sevelamer was derived from the indicative price offered by the drug manufacturer to the MOH whereas the cost of CaCO3 was obtained from the public sector cost of generic CaCO3 in the 3rd quarter of 2013 [25].

The cost of haemodialysis (HD) and continuous ambulatory peritoneal dialysis (CAPD) were obtained from a previous study of the MOH dialysis program conducted in 2001, adjusted to 2013 values by applying general inflation rates obtained from the International Monetary Fund [26, 27]. Dialysis costs were annualized assuming three HD sessions per week (156 HD sessions per year) and daily use of CAPD (365 CAPD days per year) according to the dialysis practice in Malaysia. The base case analysis inputs are listed in Table 2.

Currency conversions from RM to US$ values presented in this study were calculated using the exchange rate on 31 December 2013 of $1 to RM3.28 as the index year of study was 2013 [28].

### 2.5. Analysis

The model simulated the costs and outcomes over the lifetime of the entire cohort from initiation of CBB therapy to dialysis and/or death. Future costs and QALYs were discounted at 3% per annum to the reference year as recommended by the Malaysian Pharmacoeconomics Guidelines [29]. Analysis was also performed using life years (LY) as health outcome.

We performed scenario sensitivity analysis by varying variables as recommended by guidelines and for key variables that may possibly change the conclusions that are drawn from the base case analysis. The variables that were analysed were discount rate, time horizon, drug dose, utility while on dialysis, and HD cost. As well, a probabilistic sensitivity analysis (PSA) was performed over 10,000 simulations to capture the uncertainty in several parameters in the model, namely, hazard ratios of survival and inception of dialysis, daily doses, and unit costs of sevelamer and CaCO3.

### 2.6. Assessment Criteria

According to the WHO guidelines, a treatment is cost-effective if the incremental cost-effectiveness ratio (ICER) is below a maximum threshold of 3 times GDP per capita for a country when comparing one treatment against another [30]. The ICER, in this case, compares the cost and QALYs gained from treatment with sevelamer relative to CaCO3 [19, 29, 31, 32]. The ICER was calculated using the following formula:

(1) $$\begin{array}{c} {ICER}_{sevelamer}=\frac{{Cost}_{sevelamer}-{Cost}_{{CaCO}_{3}}}{{QALY}_{sevelamer}-{QALY}_{{CaCO}_{3}}} \end{array}$$

Based on the IMF's projected GDP per capita for Malaysia of RM33,132 in 2013, treatments with ICER below RM33,132 per QALY would be considered highly cost-effective, ICERs from RM33,132 to RM99,395 could be considered cost-effective while ICERs exceeding RM99,395 are not cost-effective [27, 30].

## 3. Results

### 3.1. Base Case Analysis

As shown in Table 3 the lifetime cost of treatment with sevelamer was higher at RM159,901 ($48,750) compared to RM77,139 ($23,518) per patient on CaCO3. Yet, treatment with sevelamer gained more life years and QALY than CaCO3. In terms of life years, 6.37 years were gained on sevelamer compared to 4.25 years on CaCO3, whereas in terms of QALYs, sevelamer gained 5.27 versus 3.54 QALY gained using CaCO3. Hence, the ICER of treatment with sevelamer relative to CaCO3 was RM39,050 ($11,906) per LY gained and RM47,679 ($14,536) per QALY gained.

At RM47,679 per QALY gained, the ICER of treatment with sevelamer was between one to two times the estimated current Malaysian GDP per capita of RM33,132 and RM66,263, respectively. Based on WHO cost-effectiveness threshold, sevelamer would be considered cost-effective compared to CaCO3 for the treatment of hyperphosphatemia in CKD patients in Malaysia.

### 3.2. Scenario Sensitivity Analysis

In the scenario sensitivity analysis, the ICER of sevelamer compared to CaCO3 varied from RM14,407 to RM 65,210 per QALY gained, with the ICER being most sensitive to varying the cost of sevelamer (due to higher assumed daily dose of sevelamer), HD cost, and the time horizon. The results of scenario sensitivity analyses are shown in Table 4.

Assuming a high sevelamer dose of 4.8g per day would increase the ICER to RM65, 210 per QALY gained whereas assuming higher HD cost of RM350 per procedure would increase the ICER to RM 58,084 per QALY gained. On the other hand, excluding the cost of dialysis in the extended lifetime of the patient would reduce the ICER to RM 14,407. Overall, none of the one-way sensitivity scenarios exceeded the two times GDP per capita threshold of RM66,263 per QALY gained and sevelamer remained cost-effective. Reducing study duration to a 3-year time horizon lowered the ICER and sevelamer becomes highly cost-effective at RM25,438 per QALY gained.

### 3.3. Probabilistic Sensitivity Analyses

**Figure 3:**
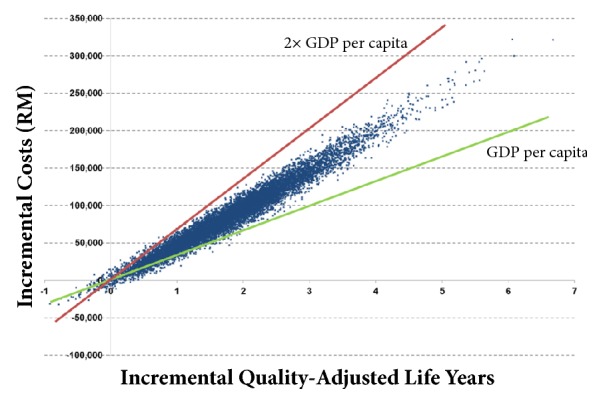
Scatter plot of ICER values generated from probabilistic sensitivity analysis.

Figure 3 illustrates the results of PSA in the form of an ICER scatterplot, which shows that, through 10,000 PSA simulations, the ICER values clustered in a narrow range around the base case ICER value of RM47,679 per QALY gained and between one and two times GDP per capita.

**Figure 4:**
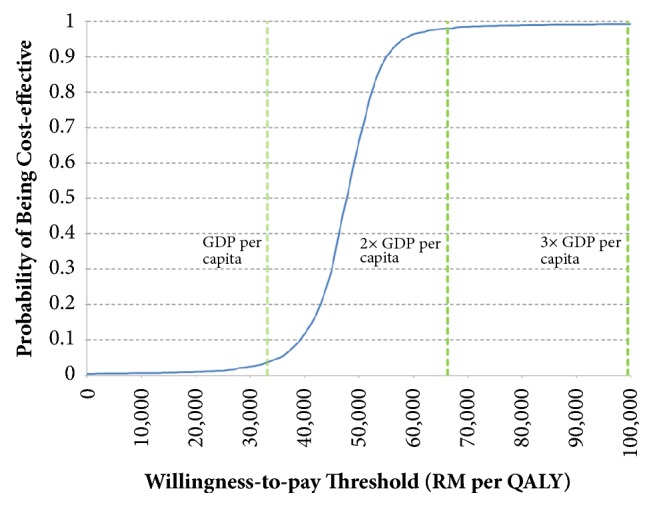
Cost-effectiveness acceptability curve.

The cost-effectiveness acceptability curve shown in Figure 4 reaffirmed that in most simulations, the ICER would be between one and two times GDP per capita. The results of PSA showed that 98.9% of simulations generated ICERs below two times GDP per capita. Based on the PSA results, sevelamer would very likely be cost-effective for the treatment of hyperphosphatemia in CKD-ND patients in Malaysia as it was unlikely that the ICER would exceed the WHO cost-effectiveness threshold of three times GDP per capita.

## 4. Discussion

Our results are consistent with the earlier studies which demonstrated cost-effectiveness of sevelamer in UK and Italy [17, 18]. The ICER is less than twice the GDP per capita for the country.

Our study has a number of strengths. To our knowledge, this study is the first attempt at an evaluation of the relative cost and benefits of sevelamer and CaCO3 in a predialysis population in a developing country setting. Secondly, we utilized Malaysian costs and utility data as inputs where possible. Thirdly, sensitivity analyses were conducted with both scenario and probabilistic sensitivity analyses. The PSA predicted that just 0.4% of simulations would exceed the WHO cost-effectiveness threshold, reinforcing the robustness of the base case findings that sevelamer is cost-effective.

We also note several limitations to the study. Firstly, the analysis was performed using a global cost-effectiveness decision model which comprised only three health states and was based on data from the INDEPENDENT-CKD trial [16]. There was also limited flexibility for customization and long-term efficacy which was based on 36-month follow-up data extrapolated to lifetime by regression modelling. However, as an independent study with a randomized, controlled trial design, it provided a possibly less biased comparative efficacy data from a matched population and remains the best available data in the absence of other head-to-head comparisons of sevelamer and CaCO3 in the local setting.

This study was technically designed to assess cost-effectiveness against the WHO threshold, but it may also be useful to compare our result against previous funding decisions by the Malaysian MOH. Of relevance is a comparison to haemodialysis treatment provided by MOH which has been estimated to cost RM33,642 per annum in 2001 in a previous study [26]. Factoring for inflation and exchange rate changes since 2001, we estimated the current cost of haemodialysis to be RM44,138 per patient per year [26, 28], which is slightly higher than the ICER of sevelamer in the present study at RM39,050 per LY. This means that increasing expenditure on a medication like sevelamer could prevent patients from going on to dialysis, which is a favourable clinical outcome as well as save costs through over the lifetime of patients. Beyond being a good clinical outcome, delay of dialysis treatment would also allow patients with early renal insufficiency to lead better quality and healthier lives which can be defined as priceless to an individual patient. From a societal point of view, renal patients who are not on dialysis have greater potential to contribute fully to the society through their familial, social, and economic contributions.

## 5. Conclusions

In conclusion, our analysis indicates that sevelamer can be a cost-effective treatment for hyperphosphatemia in Malaysian CKD-ND patients compared to CaCO3 at an ICER of RM47,679 per QALY gained. Results of sensitivity analyses did not substantially differ from the base case results and indicated that the base case results were robust and did not exceed the CE threshold of three times GDP per capita per QALY gained. Further studies incorporating long-term efficacy data, local utilities, cardiovascular disease effects, and adverse events may improve the precision of the results, but we anticipate it would be unlikely to change the overall conclusion. Our results for Malaysia suggest that sevelamer may be cost-effective in middle-income countries. This study finds that sevelamer is potentially cost-effective compared to CaCO3, for the treatment of hyperphosphatemia in predialysis CKD III-V. We propose that sevelamer should be an option in the treatment of Malaysian predialysis patients with hyperphosphatemia, particularly those with high calcium load. However, definitive conclusions about the cost-effectiveness of sevelamer should be confirmed through individual country-level studies incorporating local data.

## Figures and Tables

**Table 1:** INDEPENDENT-CKD trial patient characteristics.

Patient characteristics	Sevelamer	CaCO3
Subjects (N)	107	105
Mean age (years)	57.4	58.5
Males (%)	61	61
CKD disease stage	Stage 3–4	Stage 3–4
Diabetes (%)	27	29
Hypertension (%)	72.9	76.1
Baseline creatinine clearance (CCr) (ml/min)	31.7	32.7
Baseline phosphorus (mg/dl)	4.82	4.87
Baseline calcium (mg/dl)	9.0	8.8
Baseline parathyroid hormone (PTH) (pg/ml)	200	188

**Table 2:** Base case input values.

Parameter	Sevelamer	CaCO3	Unit	Source
Population characteristics				
Mean age at baseline	57.9	57.9	year	Di Iorio 2012
Baseline serum phosphate level	4.82	4.87	mg/dl	Di Iorio 2012
CKD stage 3	50	50	%	Assumption derived from Di Iorio 2012
CKD stage 4	50	50	%	Assumption derived from Di Iorio 2012

Drug treatment				
Sevelamer dose	2.184	n/a	g/day	Di Iorio 2012
CaCO3 dose	n/a	2.950	g/day	Di Iorio 2012
Drug cost	5.0069	0.0871	RM/g	Sevelamer cost: Sanofi, CaCO3 cost: IMS 2013

Efficacy				
Survival up to 36 months	Figure 2	Figure 2		Modelled from INDEPENDENT-CKD data
Dialysis initiation up to 36 months	Figure 3	Figure 3		Modelled from INDEPENDENT-CKD data
Long-term survival	Figure 2	Figure 2		Modelled from INDEPENDENT-CKD data
Long-term dialysis initiation	Figure 3	Figure 3		Modelled from INDEPENDENT-CKD data

Utility				
Pre-dialysis CKD utility	0.86	0.86		Gorodetskaya 2005; weighted average of utility in stage 4 and 5 CKD
Dialysis utility	0.8	0.8		Bavanandan 2010

Dialysis				
Proportion of patients on HD	88	88	%	21st MDTR 2013
Proportion of patients on CAPD	12	12	%	21st MDTR 2013
HD cost	259.09	259.09	RM/ session	Hooi 2005, IMF 2013
CAPD cost	99.04	99.04	RM/day	Hooi 2005, IMF 2013

Base case modelling parameters				
Cohort size	1000	1000	patient	Assumption
Time horizon	60	60	year	Assumption
Discount rate on costs	3	3	%	Malaysian PE Guidelines 2012
Discount rate on outcomes	3	3	%	Malaysian PE Guidelines 2012

**Table 3:** Base case results (3% discount on costs and outcomes).

Treatment	Lifetime cost per patient (RM)	Effect (LY)	ICER (Cost per LY gained)	Effect (QALY)	ICER (Cost per QALY gained)
CaCO3	77,139	4.25	-	3.54	-
Sevelamer	159,901	6.37	-	5.27	-
Incremental	82,763	2.12	39,050	1.74	47,679

**Table 4:** Scenario sensitivity analyses results.

No.	Variable	Base case input value	Sensitivity input value	ICER (Cost (RM) per QALY gained)
1a	Undiscounted cost and outcomes	3%	0%	48,033
1b	5% discount rate	3%	5%	47,419
2a	3-year time horizon	60 years	3 years	25,438
2b	10-year time horizon	60 years	10 years	47,586
3a	High sevelamer dose	2.184g	4.8g	65,210
3b	High CaCO3 dose	2.95g	7.5g	47,325
4	Low dialysis utility	0.8	0.72	51,086
5a	Low HD cost	RM259.09	RM197.48	40,627
5b	High HD cost	RM259.09	RM350.00	58,084
6	Exclude dialysis cost	RM259.09	0	14,407

## Data Availability

The data supporting the findings of this study are stated clearly and available in the manuscript.
